# A bibliometrics and visualization analysis of ropivacaine research from 2000 to 2023

**DOI:** 10.3389/fmed.2024.1465308

**Published:** 2024-10-02

**Authors:** Jian Zhang, Ye Liu, Xiyao Gu, Jing Chai

**Affiliations:** ^1^Department of Anesthesiology, International Peace Maternity and Child Health Hospital, School of Medicine, Shanghai Jiaotong University, Shanghai, China; ^2^Shanghai Key Laboratory of Embryo Original Diseases, Shanghai, China; ^3^Shanghai Municipal Key Clinical Specialty, Shanghai, China; ^4^Department of Anesthesiology, Renji Hospital, Shanghai Jiao Tong University School of Medicine, Shanghai, China; ^5^Key Laboratory of Anesthesiology (Shanghai Jiao Tong University), Ministry of Education, Shanghai, China; ^6^Department of Anesthesiology, Yantai Affiliated Hospital of Binzhou Medical University, Yantai, China

**Keywords:** ropivacaine, web of science, bibliometric analysis, Citespace, VOSviewer, co-citation analysis

## Abstract

**Aim:**

Bibliometric and data visualization methods were used to identify the current status, key areas, and emerging frontiers in ropivacaine research.

**Methods:**

We conducted a comprehensive search of the Web of Science database for publications related to ropivacaine published from 2000 to 2023. The publication types were limited to original articles and reviews. We utilized CiteSpace, VOSviewer, and the online bibliometric platform^1^ to visualize and analyze the collected data.

**Results:**

A total of 4,147 publications related to ropivacaine were identified, with a consistent growth in annual publications over time. The United States emerged as the most influential country in the field of ropivacaine research, and ranked first in the annual number of publications until 2014. China surpassed the United States in the number of publications for the first time in 2015 and has remained in first place ever since. Of all the research institutions in the field of ropivacaine, University of Copenhagen in Denmark exhibited the highest impact. Brian M. Ilfeld and Casati A were identified as the most influential authors. The leading researchers in this field primarily focused their publications on continuous nerve blocks for postoperative analgesia and ultrasound-guided nerve block techniques. An analysis of reference co-citation clustering revealed 18 distinct research clusters, with current hotspots including erector spinae plane block, dexmedetomidine, quadratus lumborum block, labor analgesia, and mitochondrial respiration. Additionally, keywords analysis indicated that “dexmedetomidine as an adjuvant in nerve blocks” currently represents a research hotspot in the field of ropivacaine.

**Conclusion:**

This bibliometric analysis provides a comprehensive overview of the research landscape in ropivacaine. It reveals research trends in this field and emerging areas for future investigations. Notably, the application of ropivacaine in nerve blocks is a prominent focus in current research, with a particular emphasis on its combination with dexmedetomidine.

## 1 Introduction

Ropivacaine is the first pure levorotatory long-acting amide local anesthetic, which possesses high pKa and low-fat solubility. It blocks the flow of sodium ions into the cell membrane of neurons, thereby leading to a reversible nerve block and inhibiting impulse conduction through nerve fibers. Ropivacaine blocks Aδ and C nerve fibers responsible for transmitting pain signals (Aδ and C fibers) but spares those involved in motor function (Aβ fibers) (1, 2). Due to its robust efficacy (3), negligible motor block (2), and minor risk of toxic effects on the central nervous system and the heart (4), ropivacaine has become an ideal choice for anesthesia (5), postoperative analgesia (6), and labor analgesia (7).

Given these unique characteristics, ropivacaine has attracted increasing attention among scholars. However, the rapid growth in the number of publications has made it progressively challenging for researchers to stay abreast of the latest developments. Although systematic reviews offer invaluable insights from specific angles of ropivacaine research, they rarely provide comprehensive data on numerical growth trends, the contributions of countries, institutions, and authors, and future research hotspots (8, 9). Early-career researchers need to gain an overview analysis of the knowledge structure and current hotspots within a specific field (10, 11). Therefore, bibliometric analysis has become an increasingly favorable method for obtaining these parameters.

Bibliometrics adopts mathematical and statistical methods to analyze and interpret previous studies. It is primarily used to assess the production, citation patterns, and trends of literature in the fields of science, technology, and other academic fields. The goals of bibliometrics can help scholars and researchers assess the impact of research results, identify patterns of research cooperation and communication, elucidate the research dynamics in specific fields, and guide researchers for future research (11). With the development of information technology, the visualization of bibliometric data has become a reality. Various bibliometric tools, such as CiteSpace (12) and VOSviewer (13), have been widely employed in various medical fields, including neurology (14, 15), oncology (16), and endocrinology (17). In this study, we utilized bibliometric tools to visualize and interpret the findings in the realm of ropivacaine research, shedding light on future trends in this field.

## 2 Materials and methods

### 2.1 Data source and search strategy

We conducted a comprehensive search using the Web of Science Core Collection (WoSCC) database (18, 19), specifically the Science Citation Index Expanded (SCI-E) edition. The following search strategy was used: [Title] OR [abstract] OR [author keyword] = [ropivacaine] OR [naropin] OR [naropeine]. We searched the literature from January 1, 2000 to December 31, 2023, and the publication type was limited to the categorization of “article” and “review” in the WoSCC. Literature search and data downloads were done on a single day, March 2, 2024, to minimize bias arising from database updates. The detailed information of references’ data downloaded were saved in .txt style for further importing. XG and JC reviewed all the references list to ensure they were related to the topic. We did not find duplicated references under the current search strategy.

### 2.2 Data processing

The data, comprising full records and cited references, were exported for subsequent analyses. CiteSpace (Version 6.2.R4, 64-bit, Drexel University, Philadelphia, PA, United States), VOSviewer (Version 1.6.20, Leiden University, Netherlands), and an online bibliometric platform (see text footnote 1) were used for further analyses.

1. CiteSpace: CiteSpace, as a widely recognized bibliometric tool, can unveil the evolutionary path of a field, detect research hotspots, and predict future directions for development (12). In the current study, CiteSpace was used to visualize data about institutions, authors, and references.
2. VOSviewer: Developed by Professor van Eck and Waltman, VOSviewer is another commonly used bibliometrics software (13). We employed VOSviewer to visualize keywords.
3. Online bibliometric platform (see text footnote 1): We utilized this online platform to measure annual publication trends within the 10 most productive countries/regions and explore inter-state cooperation.

We employed these bibliometric tools to comprehensively analyze and visualize data related to ropivacaine research, thus providing invaluable insights into trends and dynamics in the target field.

## 3 Results

### 3.1 Publication outputs and citation trends

**Figure 1:**
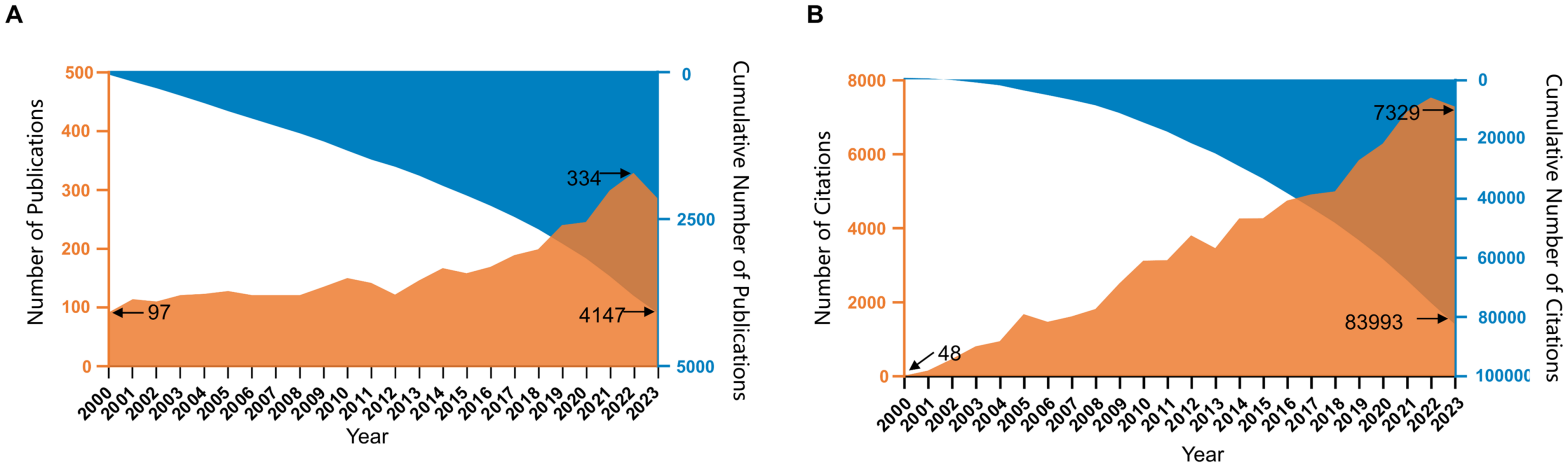
**(A)** The annual number of publications in ropivacaine research from 2000 to 2023. **(B)** The annual citations of ropivacaine research publications from 2000 to 2023.

Quantity changes in publications and citations over a period of time directly reflect the evolving trends in scientific knowledge within a particular field. In total, 4,147 publications were included in this analysis, including 4,016 original articles and 131 reviews. The annual distribution of ropivacaine research-related publications is shown in Figure 1A. Although we observed occasional fluctuations in the number of annual publications, the overall trend was upward, peaking in 2022 (334 publications in 2022 vs. 97 publications in 2000, indicating a 3.44-fold increase) (Figure 1A). Furthermore, these publications possessed 83,993 citations (67,134 citations after excluding self-citations), equal to 20.25 citations per paper. The annual distribution of citations exhibited a linearly ascending pattern (*R*^2^ = 0.981) (Figure 1B). The concurrent rise in both publications and citations emphasizes the growing interest and significance of ropivacaine research.

### 3.2 Basic knowledge structures of ropivacaine field

#### 3.2.1 Analysis of most prolific countries/regions

**Figure 2:**
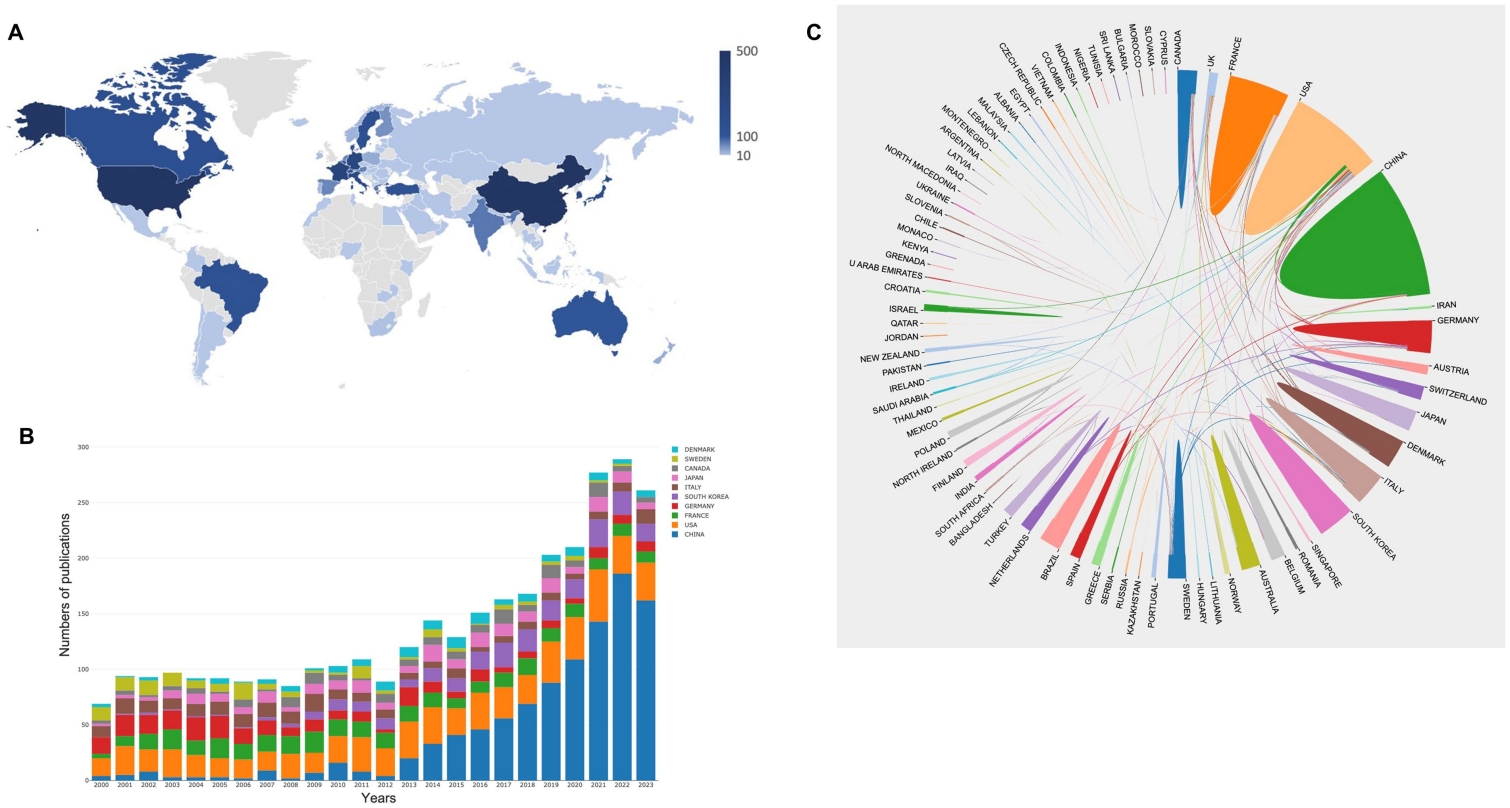
**(A)** A world map depicting the contribution of each country/region based on publication counts. **(B)** The annual number of publications in the top 10 most productive countries from 2000 to 2023. **(C)** International collaboration analysis among different countries/regions.

Figure 2A represents a world map showing the contributions of each country, with darker colors representing more publications. Publications came mainly from countries/regions in North America, Western Europe, and East Asia (Figure 2A).

Specifically, China ranked first in terms of the number of publications in the field, contributing 1,005 papers, followed by the United States with 650 papers (Table 1). The two countries were far ahead of other studies (Table 1). H-index (h) is defined as the number of publications for an individual each accruing at least h citations. It is used to assess the quality and quantity of publications from countries, journals, or authors (20). In the top 10 productive countries, the United States (68) ranked first with the highest h-indices, followed by France (47), Canada (40), and Sweden (40). This metric might have been affected by the time interval, particularly among recent entrants in this field who have not yet obtained sufficient citations. Until 2014, the United States led in terms of the number of publications (Figure 2B). However, China has exhibited rapid growth since 2012. In 2015, China surpassed the United States for the first time, maintaining the top position thereafter (Figure 2B). This trend suggests that China’s h-index is likely to increase in the near future.

**Table 1:** Top 10 countries/regions in terms of publications for ropivacaine.

Ranking	Country/Region	Publications	h-index	ACI
1	PR China	1,005	36	8.44
2	United States	650	68	29.35
3	France	312	47	24.47
4	Germany	270	37	20.43
5	South Korea	237	30	13.34
6	Italy	223	39	23.26
7	Japan	195	30	18.08
8	Canada	151	40	35.87
9	Sweden	137	40	33.31
10	Denmark	130	39	36.79

Average citations per item (ACI) is another metric that reflects the value of a paper and its contribution to the scientific community. ACI indicates the average number of citations per paper. Table 1 illustrates that the top five countries/regions with the highest ACI were Denmark (36.79), Canada (35.87), Sweden (33.31), the United States (29.35), and France (24.47). In contrast, countries such as China (8.44), South Korea (13.34), and Japan (18.08) had significantly lower ACI values compared to their counterparts (Table 1). Therefore, in addition to increasing the number of publications, these countries should focus on the quality of their publications.

Figure 2C shows the international cooperation among different countries/regions. A thicker line between two countries indicates stronger cooperation. The line thickness between two countries/regions reveals the degree of cooperation, and a thicker line between two countries reveals more cooperation (Figure 2C). The United States exhibited the most collaboration with other countries/regions. Overall, cooperation between countries was low and limited to North America and Europe. International cooperation needs to be strengthened.

#### 3.2.2 Analysis of most productive institutions

More than 3,000 institutions have contributed to the field. A comprehensive breakdown of publication counts, h-index, and ACI for the top 10 most prolific institutions is provided in Table 2. The United States and China each accounted for three, with the remaining four institutions being from Denmark, France, Canada, and England. University of Copenhagen topped the list with 104 publications, followed by University of California System with 73 publications and Assistance Publique Hopitaux Paris Aphp with 72 publications. Regarding quantitative indicators, such as h-index and ACI, the University of Copenhagen had the highest h-index of 35, followed by University of California System (29) and Astrazeneca (28). University of Toronto (40.37), University of Copenhagen (36.14), and Astrazeneca (35.37) were the three institutions with the highest ACI values. Notably, the three institutions from China exhibited the lowest h-index and ACI. Further analysis revealed that the majority of articles from these institutions were published after 2010, with a significant increase after 2015 (Figure 2B). The h-index and ACI of these articles are likely to increase in the future. As mentioned in previous studies, citations or ACI may not fully represent the effect of scientific effort or effectively exhibit the effect of an individual or an institution (21).

**Table 2:** Top 10 institutions in terms of publications for ropivacaine.

Ranking	Institution	Country/Region	Publications	h-index	ACI
1	University of Copenhagen	Denmark	104	35	36.14
2	University of California System	United States	73	29	31.15
3	Assistance Publique Hopitaux Paris Aphp	France	72	25	28.39
4	University of Toronto	Canada	57	26	40.37
5	Zhejiang University	PR China	55	14	8.38
6	University of California San Diego	United States	54	23	32.3
7	Capital Medical University	PR China	53	12	7.62
8	Sichuan University	PR China	51	11	6.35
9	Pennsylvania Commonwealth system of higher education (PCSHE)	United States	48	22	32.88
10	Astrazeneca	England	46	28	35.37

**Figure 3:**
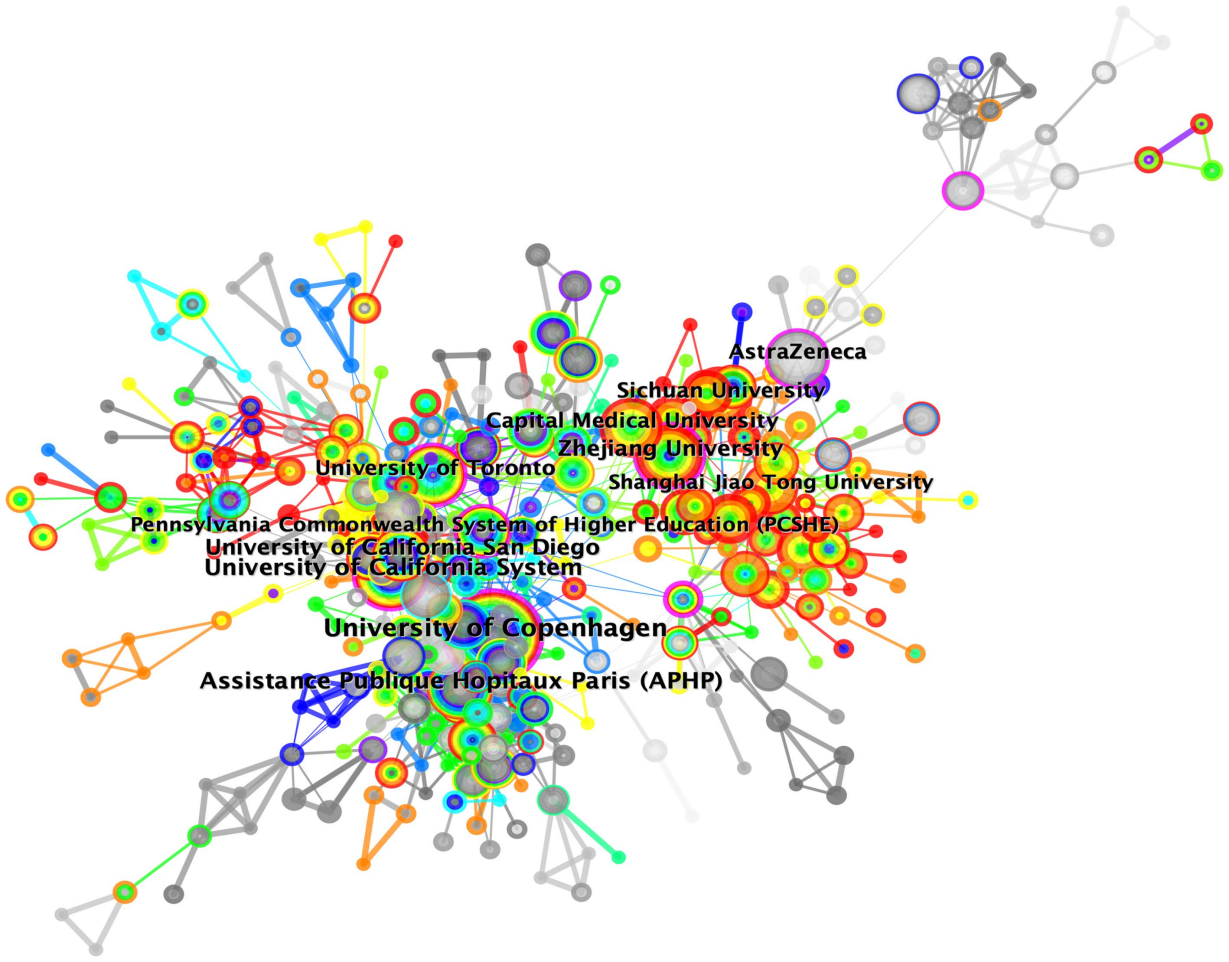
Visualization map of institution cooperation generated by CiteSpace software.

Collaboration between countries and institutions is widely recognized as an important means to improve the quality and productivity of research. We used CiteSpace software to conduct institutional cooperation analysis. The density value (0.01) substantiated cooperation between institutions (Figure 3). Betweeness centrality (BC) serves as an indicator of node centrality, reflecting the importance of a node in the network. Typically, nodes with a BC value of more than 0.1 hold pivotal positions (22), connecting a significant number of nodes. They are closely connected to other nodes, and are often identified as hubs, denoted by purple rings in the figure. Eight institutions, including Harvard University (0.23), University of Copenhagen (0.21), Pennsylvania Commonwealth System of Higher Education (PCSHE) (0.19), AstraZeneca (0.17), University of California System (0.14), University of Toronto (0.13), Ruprecht Karls University Heidelberg (0.12), and Zhejiang University (0.11), had a BC value more than or equal to 0.1, suggesting their leading position in this field. All in all, it is needed to resolve academic barriers, enhance international cooperation, and encourage communication between different research institutions and teams.

#### 3.2.3 Analysis of most influential authors

The number of scientific publications authored by an individual can serve as a proxy for their research activity and contribution to the field. In the field of ropivacaine research, more than 15,000 authors have participated in the publication of 4,147 papers. Ilfeld et al. published 57 papers (Table 3). The number was significantly higher than that published by other authors, and he held the highest h-index (Table 3). Notably, six of his publications have garnered over 100 citations, all of which were studies of continuous nerve block for postoperative analgesia (23–28). Casati A ranked second regarding the number of publications and h-index. Two of his articles have been cited more than 100 times, both of which were about nerve block under ultrasound-guided technique (29, 30). Guido Fanelli, the third most prolific author and the third author in terms of h-index, has closely collaborated with Casati A; thus, their publications significantly overlapped. Fanelli and Casati A shared two publications with over 100 citations (29, 30).

**Table 3:** Top 10 authors in terms of publications for ropivacaine.

Ranking	Author	Publications	h-index	ACI
1	Ilfeld, Brain M.	57	32	45.98
2	Casati, A	37	24	41.57
3	Fanelli, Guido	33	21	40.18
4	Mariano, Edward R.	29	18	33.24
5	Capdevila, Xavier	27	19	42.44
6	Borgeat, A.	26	21	54.88
7	Marhofer, Peter	25	19	49.88
8	Dahl, Jorgen B.	24	18	60.79
9	Loland,Vanessa J.	23	19	41.65
10	Sessler, Daniel lra	22	17	45.59

Based on ACI, there were two authors with ACI over 50, reflecting the high quality of their publications. Dahl, Jorgen B. had an ACI of 60.79, far ahead of others. He published five articles that have been cited more than 100 times. His article entitled “Adductor Canal Block Versus Femoral Nerve Block for Analgesia After Total Knee Arthroplasty A Randomized, Double-blind Study” has been cited 234 times since its publication in 2014. In their study, adductor canal block and femoral nerve block were conducted using ropivacaine for postoperative analgesia. Adductor canal block preserved quadriceps muscle strength better than femoral nerve block, without a significant difference in postoperative pain (31). His other four articles that have been cited more than 100 times were also studies on adductor canal block, indicating that his research focused on adductor canal block (32–35). Borgeat A. had the second-highest ACI. He focused on postoperative analgesia after shoulder surgery (31, 36, 37).

**Figure 4:**
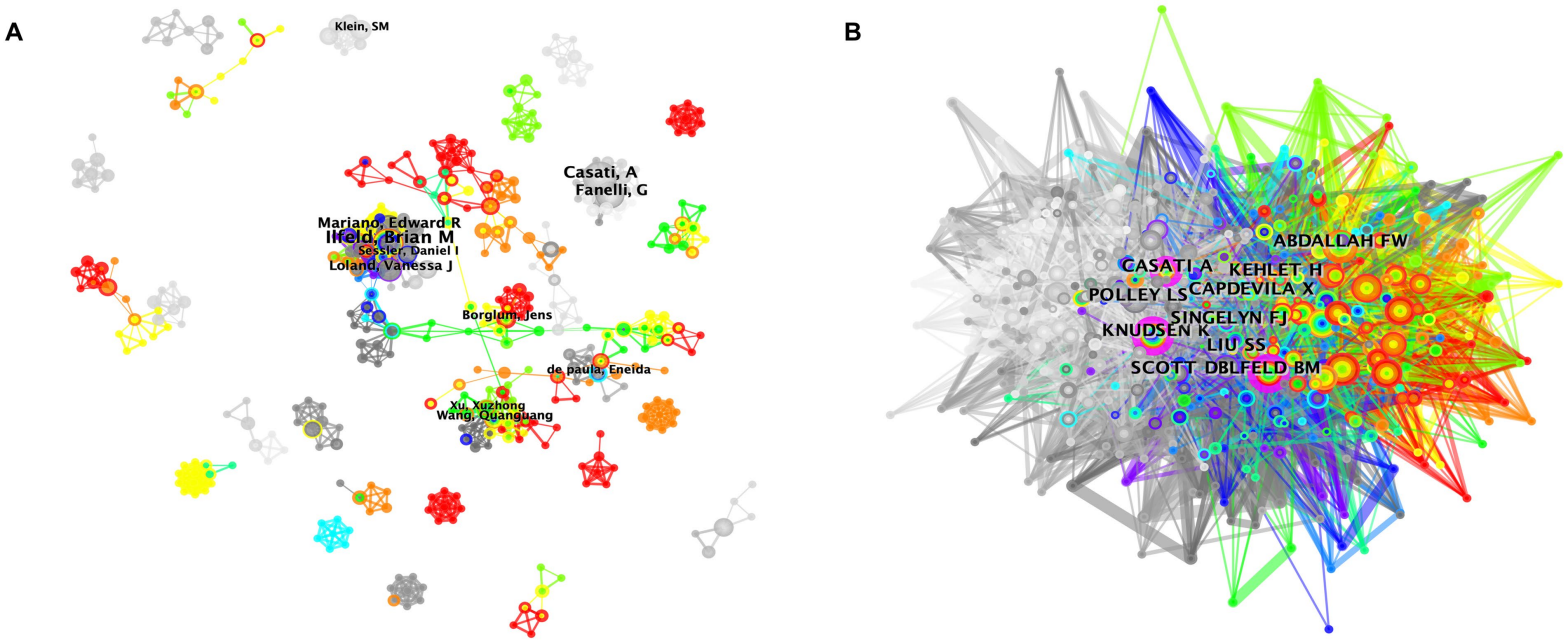
**(A)** Overlay visualization map of author co-authorship analysis generated by VOSviewer software. **(B)** Visualization map of author co-citation analysis by using CiteSpace software.

Collaboration among scholars with diverse research priorities can foster communication and productivity within a particular research subject. Co-authorship analysis can help researchers identify researchers in existing research directions, find potential partners in research fields, and reduce research detcorners, thereby improving research efficiency. Figure 4A presents an overlay visualization map of author co-authorship analysis generated by CiteSpace (Figure 4A). The map was relatively scattered, with a low density (0.004), and no author had a BC value greater than or equal to 0.1, indicating that communication and collaboration were uncommon between scholars in this field.

Co-citation analysis is used to assess the relationship between two authors or publications appearing together in the reference list of a third document (38). Author co-citation analysis can help identify key authors in a co-citation network in a specific field. Generally, frequently cited authors possess a greater impact. Figure 4B, generated by CiteSpace, revealed relatively concentrated co-citation patterns, with three authors showing a betweeness centrality (BC) of more than 0.1. The BC value of K. Knudsen was 0.15 due to his paper entitled “Central Nervous and Cardiovascular Effects of i.v. Infusions of Ropivacaine, Bupivacaine, and Placebo in Volunteers” published in 1997 (39). They found that ropivacaine showed a higher tolerated dose and unbound plasma concentration based on the shift in dose–response and concentration–response curves for CNS symptoms. Furthermore, CNS symptoms and cardiovascular changes, such as depression of conduction and diastolic function, were less pronounced with ropivacaine compared to bupivacaine. The other two authors were Casati A. (0.14) and Brian M. Ilfeld (0.11). These results indicated that Brian M. Ilfeld and Casati A. were the most influential authors in the field of ropivacaine research, both in terms of publication volume, publication quality, and the impact of their articles.

### 3.3 An overview of research hotspots and frontiers

#### 3.3.1 Analysis of highly-cited studies

Citation analysis is a cornerstone in bibliometric studies. Although some debates remain toward the significance of citation rates (40), it is widely accepted that the number of citations can largely reflect the impact of publications. Higher citation frequencies generally indicate a higher academic (41). We summarized the 10 most cited papers on ropivacaine (Table 4). Among them, eight were original articles and two were systematic reviews, all of which were cited more than 250 times.

**Table 4:** Top 10 co-cited references related to vascular cognitive impairment in terms of co-citations.

Citations	Title	Source	First author	Publication year
413	Local infiltration analgesia: a technique for the control of acute postoperative pain following knee and hip surgery—a case study of 325 patients.	Acta Orthopaedica	Kerr, Dennis R	2008
397	The analgesic efficacy of transversus abdominis plane block after cesarean delivery: a randomized controlled trial.	Anesthesia and Analgesia	McDonnell, John G	2008
358	Efficacy of continuous wound catheters delivering local anesthetic for postoperative analgesia: a quantitative and qualitative systematic review of randomized controlled trials.	Journal of the American College of Surgeons	Liu, Spencer S.	2006
310	The transversus abdominis plane block provides effective postoperative analgesia in patients undergoing total abdominal hysterectomy.	Anesthesia and Analgesia	Carney, John	2008
281	A multimodal analgesia protocol for total knee arthroplasty—a randomized, controlled study.	Journal of Bone and Joint Surgery-American Volume	Vendittoli, PA	2006
277	Different pain scores in single transumbilical incision laparoscopic cholecystectomy vs. classic laparoscopic cholecystectomy: a randomized controlled trial.	Surgical Endoscopy and other Interventional Techniques	Tsimoyiannis, Evangelos C.	2010
270	The analgesic efficacy of pre-operative bilateral erector spinae plane (ESP) blocks in patients having ventral hernia repair.	Anesthesia	Chin, K. J.	2017
270	Effect of local anesthetic volume (20 vs. 5 mL) on the efficacy and respiratory consequences of ultrasound-guided interscalene brachial plexus block.	British Journal of Anesthesia	Riazi, S.	2008
268	Successful resuscitation of a patient with ropivacaine-induced asystole after axillary plexus block using lipid infusion.	Anesthesia	Litz, R. J.	2006
265	Levobupivacaine—a review of its pharmacology and use as a local anesthetic.	Drugs	Foster, RH	2000

Notably, the paper published by Dennis R. Kerr (42) was the most highly cited paper in the field with 413 citations. In their work, Kerr and colleagues introduced a multimodal technique known as “local infiltration analgesia” (LIA) for pain control after knee and hip surgery. LIA involves systematic infiltration of a mixture of ropivacaine, ketorolac, and adrenaline into tissues surrounding the surgical site. They successfully applied LIA to manage the postoperative pain of 325 patients undergoing elective hip resurfacing (HRA), primary total hip replacement (THR), or primary total knee replacement arthroplasty (TKR) between January 1, 2005, and December 31, 2006, achieving satisfactory results in controlling postoperative pain. The second most-cited paper with 397 citations, published by John G. McDonnell (43) demonstrated the efficacy of ropivacaine for abdominal transversus abdominis plane block after C-section, which led to exceptional analgesia. In third place was a review by Spencer S. (44), encompassing 44 randomized controlled trials (RCTs) for meta-analysis and 51 RCTs for qualitative analysis. This review underscored the efficacy of continuous wound catheters, showing improved analgesia, reduced opioid use and side effects, higher patient satisfaction, and shorter hospital stays.

#### 3.3.2 Reference co-citation analysis

**Figure 5:**
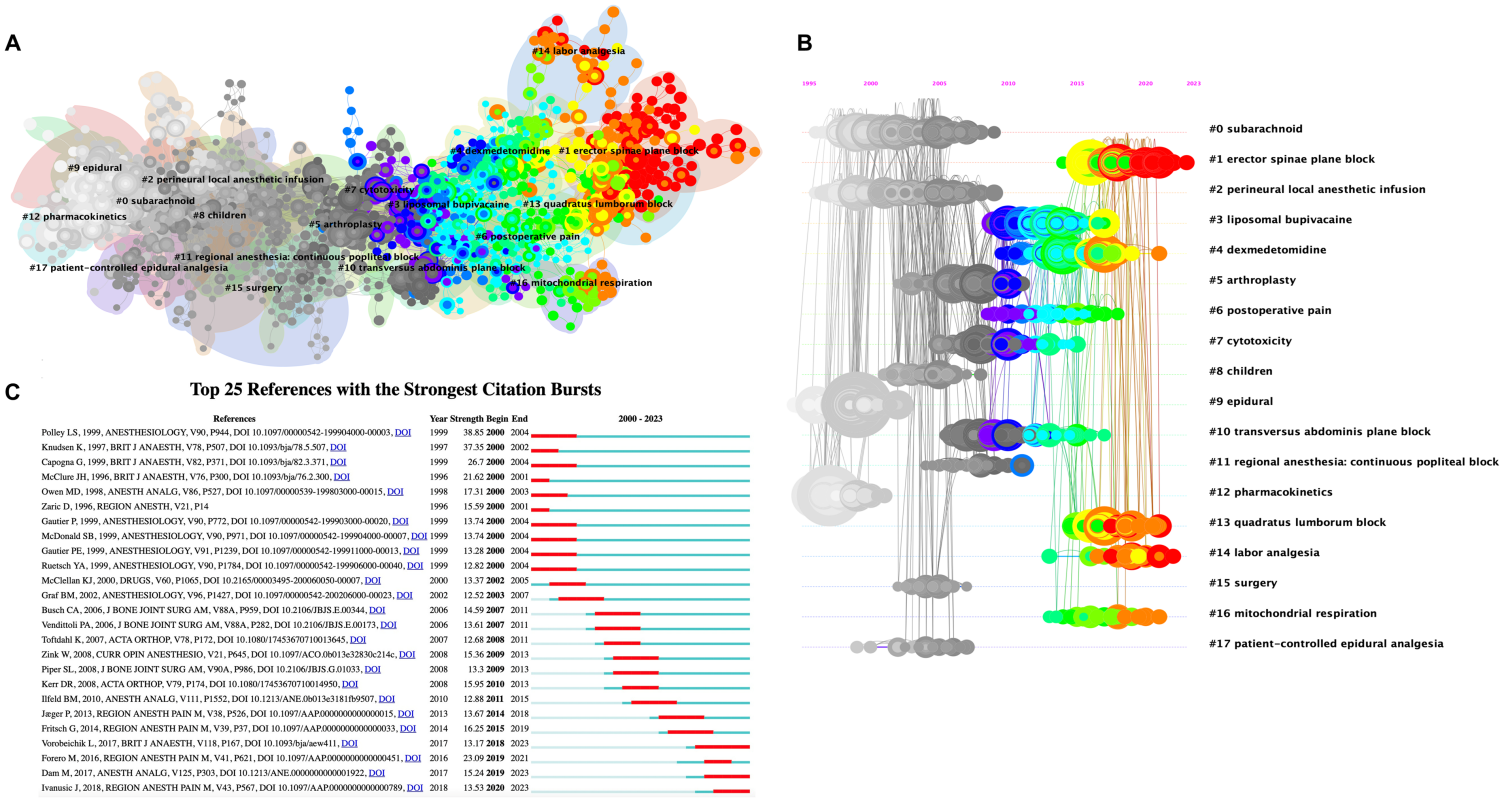
The cluster view map **(A)** and timeline view map **(B)** of reference co-citation analysis were generated by CiteSpace. **(C)** Visualization map of top 25 references with the strongest citation bursts involved in ropivacaine.

Reference co-citation analysis identified 18 major clusters, which helped us identify the changes in research clusters in the target field, and identify both clusters of landmark references and ongoing research (Figure 5A). We produced a timeline view of these clusters to trace the evolving focus over time (Figure 5B).

Except for liposomal bupivacaine (#3), toxicity (#7), pharmacokinetics (#12), and mitochondrial respiration (#16) (Figure 5), the remaining clusters were all about the use of ropivacaine in anesthesia or analgesia. Early studies focused on local infiltration anesthesia (#2), spinal anesthesia (#0), and epidural applications (#9 and #17). Its use in children (#8) and arthroplasty surgery (#5), and in combination with dexmedetomidine (#4) has attracted much attention for a period of time. With the progress and gradual application of ultrasound technology, it has been widely used in nerve block, including erector spinae plane block (# 1), transversus abdominis plane block (#10), popliteal block (#11), and quadratus lumborum block (#13). In addition, the use of ropivacaine in labor analgesia (#14) has garnered much attention. Currently, the most prominent trends include “erector spinae plane block” (#1), “dexmedetomidine” (#4), “quadratus lumborum block” (#13), “labor analgesia” (#14), and “mitochondrial respiration” (#16). An in-depth analysis of the literature in each cluster, especially the representative works in the cluster can provide invaluable insights. “Erector spinae plane block” (#1) and “quadratus lumborum block” (#13) represent investigations into relatively novel targets for nerve blocks, benefiting from the integration of ultrasound technology (45–50). “Dexmedetomidine” (#4), predominantly consisted of studies exploring the combined use of dexmedetomidine and local anesthetics, such as ropivacaine (51–53). “Labor analgesia” (#14) focused on ropivacaine for labor analgesia. Because of its satisfactory efficacy, low toxicity, and dissociation of motor block and sensory block, it is widely used in labor analgesia (54). “Mitochondrial respiration” (#16) implied that ropivacaine can induce energy loss, oxidative stress, and oxidative damage by inhibiting mitochondrial respiration (55).

#### 3.3.3 Analysis of references with citation burst

Burst detection is an algorithm developed by Kleinberg, which can effectively capture notable increases in the popularity of references or keywords within specific timeframes. This function provides an efficient means of identifying concepts or topics that have been actively discussed over a defined period. In this study, we applied burst detection to extract key references in the field of ropivacaine research. The top 25 references with the most significant citation bursts are listed in Figure 5C. In the graphical representation, the blue lines indicate the time intervals and the red segments represent the periods of reference bursts. Among these articles, the one with the most robust burst value was written by Linda S. Polley et al. (38.85) (56). In their study, they compared ropivacaine to bupivacaine for epidural analgesia during labor and found that ropivacaine was notably less potent than bupivacaine during the first stage of labor. Following closely, the reference with the second-highest burst value was published by K. Knudsen et al. (37.35), which indicated that ropivacaine can exhibit fewer central nervous system and cardiovascular effects compared to bupivacaine (39). Furthermore, while the burst in the majority of references has subsided, three references still exhibited ongoing bursts. This finding indicates that these topics continue to garner significant attention in the near future. One article investigated how dexmedetomidine can enhance the quality of anesthesia for brachial plexus blocks (52). One was a cadaveric study of the pathway of injectate spread with the transmuscular quadratus lumborum block (47). The other was a cadaveric study investigating the mechanism of action of erector spinae blockade (50). These are consistent with reference clusters #1, #4, and #13.

#### 3.3.4 Analysis of the most frequently appearing keywords

**Figure 6:**
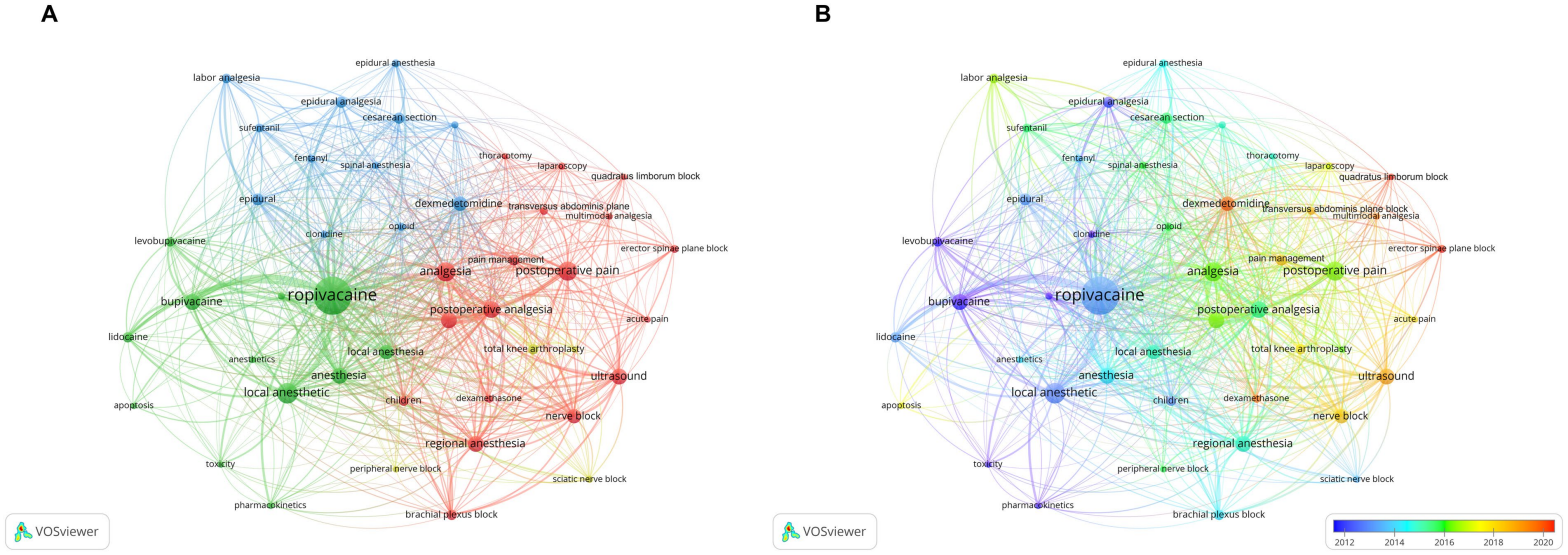
**(A)** Overlay visualization map of keywords co-occurrence analysis. **(B)** The changes in keywords from 2012 to 2023.

Keywords serve as pivotal indicators of the main topic and core content in a specific field (57). Another prevalent method of bibliometrics for identifying hot research topics is keyword co-occurrence analysis. Co-occurrence analysis is used to assess the association of keywords based on the number of documents in which they appear together (58). We analyzed author keywords extracted from 4,147 publications using VOSviewer. Forty-six author keywords with more than 30 occurrences were extracted by consolidating keywords with synonymous meanings. Table 5 lists the 20 most frequently occurring keywords. Ropivacaine, local anesthetic, postoperative pain, analgesia, and postoperative analgesia were keywords with more than 200 co-occurrences. Remarkably, there were eight keywords related to nerve block, including nerve block, brachial plexus block, erector spinae plane block, transversus abdominis plane block, sciatic nerve block, femoral nerve block, quadratus lumborum block, and peripheral nerve block. The keyword co-occurrence visualization map generated by VOSviewer (Figure 6A) showed that all keywords were grouped into four clusters with different colors. We found the following keywords from the largest cluster to the smallest cluster: postoperative analgesia, anesthesia, obstetric application, and nerve block. These common keywords primarily were about the utilization of ropivacaine in anesthesia, analgesia and nerve block and comparison to other local anesthetics and analgesics (Figure 6A).

**Table 5:** Top 10 keywords in terms of co-occurrences for ropivacaine.

Ranking	Keyword	Count	Ranking	Keyword	Count
1	Ropivacaine	1,094	11	Nerve block	167
2	Local anesthetic	314	12	Dexmedetomidine	157
3	Postoperative pain	289	13	Local anesthesia	157
4	Analgesia	281	14	Epidural	112
5	Postoperative analgesia	218	15	Epidural analgesia	101
6	Pain	198	16	Pain management	101
7	Regional anesthesia	195	17	Cesarean section	98
8	Bupivacaine	190	18	Brachial plexus block	87
9	Anesthesia	188	19	Total knee arthroplasty	81
10	Ultrasound	186	20	Lidocaine	79

Furthermore VOSviewer was used to color-cod all keywords based on their average appearance year (Figure 6B). Keywords surfacing earlier are denoted in blue while those with a more recent appearance are highlighted in red. Notably keywords like “bupivacaine,” “epidural analgesia,” and “toxicity” were prominent during the early stages of research. Conversely keywords such as “quadratus lumborum block,” “erector spinae plane block,” “dexmedetomidine,” and “dexamethasone” exhibited a more recent average appearance year. This finding aligns with the findings of our reference co-citation analysis suggesting that the use of dexmedetomidine as an adjuvant in nerve block has garnered increasing attention and currently stands as a major research focus.

## 4 Discussion

### 4.1 Primary findings

Our study employed bibliometric analysis to analyze 4,147 publications in the field of ropivacaine research between 2000 and 2023. The number of ropivacaine-related publications has significantly increased since 2000. The United States has long been a leader in terms of the quantity and quality of publications. However, China’s contributions to this field rapidly increased since 2012, and it has occupied the first place since 2015. Among research institutions, University of Copenhagen exhibited the highest publication output and quality. As for the authors, Brian M. Ilfeld and Casati A. were the most influential authors. The primary focus of ropivacaine-related studies has been on its clinical use, particularly in various methods of nerve block and ultrasound-guided nerve block. A closer look at co-cited publication clusters unveiled the dominant research themes since 2000, which included the erector spinae plane block, quadratus lumborum block, the combined use of dexmedetomidine and ropivacaine and the synergistic effects of dexamethasone and ropivacaine. Keyword analysis was also used to underscore the current hotspots in the field of ropivacaine research, emphasizing its clinical applications as the central theme in the field.

### 4.2 Results of the study in context

#### 4.2.1 The current academic situation of countries/regions and institutions on ropivacaine research

North America, Western Europe, and East Asia were the leaders in the field of ropivacaine research. Notably, China has led in terms of the quantity of publications and the United States has led in terms of the quality of publications (h-index and ACI). Since 2015, China has risen to the top of the ranking in terms of publications, suggesting China’s growing impact in the field of ropivacaine research. It may be related to China’s economic development and the widespread use of nerve block techniques. International cooperation is currently limited in the field of ropivacaine research, with collaborations primarily focused on the United States and Europe. There is a need for research institutions to gradually break down academic barriers and promote cooperation and exchange between research institutions and researchers worldwide.

#### 4.2.2 Nerve block is the most extensive clinical application of ropivacaine

Ropivacaine is an efficient local anesthetic, which can be widely used in both adults and children for epidural anesthesia, analgesia, local infiltration anesthesia, and peripheral nerve block. Notably, researchers, such as Brian M. Ilfeld and Casati A., have made significant contributions to the field. They focused on continuous nerve block for postoperative analgesia (23–28) and ultrasound-guided nerve block anesthesia (29, 30). They markedly advanced the use of ropivacaine in clinical practice. Furthermore, ropivacaine has gained attention in pediatric regional anesthesia, and Peter Marhofer confirmed its effectiveness. Particularly, it garnered much attention in ultrasound imaging of the infraclavicular brachial plexus in children (59). Nerve block anesthesia is a local technique of anesthesia, which disrupts nerve conduction by injecting local anesthetics near nerve trunks or plexuses. It has been widely adopted to induce sensory and motor block at surgical and painful sites. Our study indicated that popliteal block, transversus abdominis plane block, quadratus lumborum block, and erector spinae plane block have all attracted much attention.

Several factors contribute to the widespread use of ropivacaine for nerve block. Firstly, it inhibits sodium ion channels in neurons effectively blocking impulse conduction along nerve fibers, and producing reversible sensory and motor block. Secondly, the lower lipophilicity of ropivacaine, compared to other local anesthetics, like bupivacaine, results in reduced potency and delayed onset of action on large motor nerves. However, it more selectively blocks Aδ and C nerve fibers, allowing for sensory and motor separation. Moreover, ropivacaine exhibits a favorable safety profile with minimal toxicity to the central nervous and cardiovascular systems. This safety profile has encouraged its clinical application.

Although advancements in ultrasound-guided techniques can enhance the precision and safety of nerve blocks, there is still room for further research to unravel the mechanisms underlying sensory and motor separation after administering ropivacaine. Therefore, continuous clinical and basic studies are necessary to unlock the full potential of ropivacaine.

#### 4.2.3 The major trends and advantages of studies related to ropivacaine

Cluster analysis and burst detection analysis can be used to identify the prevailing research trends in the field of ropivacaine. The current focus of ropivacaine research revolves around its synergistic effects when combined with other drugs and the exploration of innovative nerve block points. In terms of drug combinations, dexmedetomidine has been widely used in the field of anesthesia, and the combination of dexmedetomidine and local anesthetics has received increasing attention. Notably, a clinical trial conducted in 2017 has significantly contributed to this trend (52). This trial assessed the analgesic efficacy of dexmedetomidine in combination with ropivacaine. It evaluated sensory and motor block and duration of analgesia. This trial provided high-level evidence through clinical trials supporting the analgesic effects of dexmedetomidine as an adjunct to peripheral nerve block. Consequently, ‘dexmedetomidine’ has emerged as a prominent category in cluster analysis. In recent years, the combination of dexamethasone and ropivacaine in nerve block has gained significant attention (60–62). This approach can enhance and prolong the nerve block effect of local anesthetics, minimize dosage and toxicity, improve the quality of postoperative analgesia, and reduce complications, such as postoperative nausea and vomiting. However, to ensure the safety and efficacy of combined medication, it is imperative to gain a comprehensive understanding of the mechanisms and pharmacological effects of drug interactions. This necessitates rigorous and systematic clinical and basic research efforts, contributing to the development of safer, more comfortable, and efficient strategies for anesthesia.

With the continuous advancement of ultrasound technology and the widespread use of nerve block techniques, the exploration of novel block points has become a major trend in the field of ropivacaine research. Since 2016, an influential article written by Forero has led the forefront of ongoing research by introducing a groundbreaking technique, namely the erector spinae plane block. This innovative inter-fascial plane nerve block method, utilizing ropivacaine, was showcased in four clinical cases, demonstrating its remarkable analgesic effectiveness. The publication also meticulously elucidated the anatomy of the erector spinae muscle (48). Another current focal point of research is the quadratus lumborum block, which represents a relatively recent addition to the repertoire of nerve block sites (45–50). The exploration of new nerve block points necessitates a profound understanding of anatomy, pharmacology, physiology, and pathophysiology. Furthermore, guidance techniques, like ultrasound and electrical stimulation, can facilitate these endeavors. Furthermore, ropivacaine holds promise for synergizing with nerve block techniques to achieve safer and more efficient nerve block anesthesia. Therefore, the research into new block points for nerve blocks merits extensive studies by both clinical and basic researchers. This collective effort aims to unlock the full potential of ropivacaine in achieving optimal anesthesia and analgesia outcomes.

In summary, the clinical application of ropivacaine has garnered significant attention. The pursuit of high-quality drug combinations and efficient nerve block strategies is of great importance for advancing the field of clinical anesthesia.

### 4.3 Limitations

This study had several limitations that warrant acknowledgment. Firstly, the study exclusively focused on publications from the Web of Science Core Collection database. Consequently, this study might have overlooked pertinent publications only accessible through other sources, such as Medline and Google Scholar. Secondly, our study was limited to publications available in the English language. Therefore, publications in other languages might not have been included, potentially underestimating contributions from non-English sources. Thirdly, the reliance of this study on citation analysis suggests that the impact of recently published high-quality publications might have been underestimated. These publications might not have had adequate time to receive a substantial number of citations within the specified timeframe of this analysis.

## 5 Conclusion

This comprehensive bibliometric analysis shed light on the research landscape of ropivacaine, offering novel insights into current trends and emerging hotspots. The combination of dexmedetomidine with ropivacaine and other local anesthetics for nerve block has become a major research focus. Additionally, researchers are exploring new nerve block points and investigating the synergistic effects of ropivacaine with other drugs. This analysis has provided invaluable guidance for future studies, encouraging further exploration of these promising areas.

## Data Availability

The original contributions presented in the study are included in the article/Supplementary material, further inquiries can be directed to the corresponding authors.

## References

[1] Hansen TG. Ropivacaine: a pharmacological review. Expert Rev Neurother. (2004) 4:781–91. doi: 10.1586/14737175.4.5.781 15853505

[2] McClellan KJ Faulds D. Ropivacaine: an update of its use in regional anaesthesia. Drugs. (2000) 60:1065–93. doi: 10.2165/00003495-200060050-00007 11129123

[3] Lacassie HJ Columb MO Lacassie HP Lantadilla RA. The relative motor blocking potencies of epidural bupivacaine and ropivacaine in labor. Anesth Analg. (2002) 95:204–8. doi: 10.1097/00000539-200207000-00036, PMID: 12088969

[4] Stewart J Kellett N Castro D. The central nervous system and cardiovascular effects of levobupivacaine and ropivacaine in healthy volunteers. Anesth Analg. (2003) 97:412–6. doi: 10.1213/01.ANE.0000069506.68137.F2, PMID: 12873927

[5] Boztuğ N Bigat Z Karsli B Saykal N Ertok E. Comparison of ropivacaine and bupivacaine for intrathecal anesthesia during outpatient arthroscopic surgery. J Clin Anesth. (2006) 18:521–5. doi: 10.1016/j.jclinane.2006.03.006, PMID: 17126781

[6] Zha J Ji S Wang C Yang Z Qiao S An J. Thoracic paravertebral nerve block with Ropivacaine and adjuvant Dexmedetomidine produced longer analgesia in patients undergoing video-assisted Thoracoscopic lobectomy: a randomized trial. J Healthcare Eng. (2021) 2021:1846886. doi: 10.1155/2021/1846886 PMC8443377 34540184

[7] Beilin Y Halpern S. Focused review: ropivacaine versus bupivacaine for epidural labor analgesia. Anesth Analg. (2010) 111:482–7. doi: 10.1213/ANE.0b013e3181e3a08e, PMID: 20529986

[8] Raines S Hedlund C Franzon M Lillieborg S Kelleher G Ahlén K. Ropivacaine for continuous wound infusion for postoperative pain management: a systematic review and meta-analysis of randomized controlled trials. Eur Surg Res. (2014) 53:43–60. doi: 10.1159/000363233, PMID: 25060049

[9] Xu P Zhang S Tan L Wang L Yang Z Li J. Local anesthetic Ropivacaine exhibits therapeutic effects in cancers. Front Oncol. (2022) 12:836882. doi: 10.3389/fonc.2022.836882, PMID: 35186766 PMC8851418

[10] Li T Yang A Liu G Zou S Chen Y Ni B . Status quo and research trends of Craniopharyngioma research: a 10-year bibliometric analyses (from 2011 to 2020). Front Oncol. (2021) 11:744308. doi: 10.3389/fonc.2021.744308, PMID: 34660308 PMC8516404

[11] Lu W Ren H. Diseases spectrum in the field of spatiotemporal patterns mining of infectious diseases epidemics: a bibliometric and content analysis. Front Public Health. (2022) 10:1089418. doi: 10.3389/fpubh.2022.1089418 36699887 PMC9868952

[12] Chen C Song M. Visualizing a field of research: a methodology of systematic scientometric reviews. PLoS One. (2019) 14:e0223994. doi: 10.1371/journal.pone.0223994, PMID: 31671124 PMC6822756

[13] Yeung AWK Tzvetkov NT Balacheva AA Georgieva MG Gan RY Jozwik A . Lignans: quantitative analysis of the research literature. Front Pharmacol. (2020) 11:37. doi: 10.3389/fphar.2020.00037, PMID: 32116713 PMC7020883

[14] Liu S Sun YP Gao XL Sui Y. Knowledge domain and emerging trends in Alzheimer's disease: a scientometric review based on CiteSpace analysis. Neural Regen Res. (2019) 14:1643–50. doi: 10.4103/1673-5374.255995, PMID: 31089065 PMC6557102

[15] Sabe M Pillinger T Kaiser S Chen C Taipale H Tanskanen A . Half a century of research on antipsychotics and schizophrenia: a scientometric study of hotspots, nodes, bursts, and trends. Neurosci Biobehav Rev. (2022) 136:104608. doi: 10.1016/j.neubiorev.2022.104608, PMID: 35303594

[16] Zhong D Li Y Huang Y Hong X Li J Jin R. Molecular mechanisms of exercise on cancer: a bibliometrics study and visualization analysis via CiteSpace. Front Mol Biosci. (2021) 8:797902. doi: 10.3389/fmolb.2021.797902 35096970 PMC8794585

[17] Wu M Wang Y Yan C Zhao Y. Study on subclinical hypothyroidism in pregnancy: a bibliometric analysis via CiteSpace. J Matern Fetal Neonatal Med. (2022) 35:556–67. doi: 10.1080/14767058.2020.1729731, PMID: 32106735

[18] Wáng YX Arora R Choi Y Chung HW Egorov VI Frahm J . Implications of web of science journal impact factor for scientific output evaluation in 16 institutions and investigators' opinion. Quant Imag Med Surg. (2014) 4:453–61. doi: 10.3978/j.issn.2223-4292.2014.11.16, PMID: 25525577 PMC4256244

[19] Yi F Yang P Sheng H. Tracing the scientific outputs in the field of Ebola research based on publications in the web of science. BMC Res Notes. (2016) 9:221. doi: 10.1186/s13104-016-2026-2, PMID: 27083891 PMC4832479

[20] Jeang KT. H-index, mentoring-index, highly-cited and highly-accessed: how to evaluate scientists? Retrovirology. (2008) 5:106. doi: 10.1186/1742-4690-5-106, PMID: 19032780 PMC2607307

[21] Giustini AJ Axelrod DM Lucas BP Schroeder AR. Association between citations, Altmetrics, and article views in pediatric research. JAMA Netw Open. (2020) 3:e2010784. doi: 10.1001/jamanetworkopen.2020.10784, PMID: 32687584 PMC7372320

[22] Zhang XL Zheng Y Xia ML Wu YN Liu XJ Xie SK . Knowledge domain and emerging trends in vinegar research: a bibliometric review of the literature from WoSCC. Food Secur. (2020) 9:1–33. doi: 10.3390/foods9020166, PMID: 32050682 PMC7074530

[23] Ilfeld BM Morey TE Wang RD Enneking FK. Continuous popliteal sciatic nerve block for postoperative pain control at home: a randomized, double-blinded, placebo-controlled study. Anesthesiology. (2002) 97:959–65. doi: 10.1097/00000542-200210000-00031, PMID: 12357165

[24] Ilfeld BM Morey TE Wright TW Chidgey LK Enneking FK. Continuous interscalene brachial plexus block for postoperative pain control at home: a randomized, double-blinded, placebo-controlled study. Anesth Analg. (2003) 96:1089–95. doi: 10.1213/01.ANE.0000049824.51036.EF, PMID: 12651666

[25] Ilfeld BM Morey TE Enneking FK. Continuous infraclavicular brachial plexus block for postoperative pain control at home: a randomized, double-blinded, placebo-controlled study. Anesthesiology. (2002) 96:1297–304. doi: 10.1097/00000542-200206000-00006, PMID: 12170039

[26] Ilfeld BM Duke KB Donohue MC. The association between lower extremity continuous peripheral nerve blocks and patient falls after knee and hip arthroplasty. Anesth Analg. (2010) 111:1552–4. doi: 10.1213/ANE.0b013e3181fb9507, PMID: 20889937 PMC3271722

[27] Ilfeld BM Vandenborne K Duncan PW Sessler DI Enneking FK Shuster JJ . Ambulatory continuous interscalene nerve blocks decrease the time to discharge readiness after total shoulder arthroplasty: a randomized, triple-masked, placebo-controlled study. Anesthesiology. (2006) 105:999–1007. doi: 10.1097/00000542-200611000-00022, PMID: 17065895

[28] Charous MT Madison SJ Suresh PJ Sandhu NPS Loland VJ Mariano ER . Continuous femoral nerve blocks: varying local anesthetic delivery method (bolus versus basal) to minimize quadriceps motor block while maintaining sensory block. Anesthesiology. (2011) 115:774–81. doi: 10.1097/ALN.0b013e3182124dc6, PMID: 21394001 PMC3116995

[29] Casati A Baciarello M Cianni SD Danelli G de Marco G Leone S . Effects of ultrasound guidance on the minimum effective anaesthetic volume required to block the femoral nerve. Br J Anaesth. (2007) 98:823–7. doi: 10.1093/bja/aem100, PMID: 17478453

[30] Casati A Danelli G Baciarello M Corradi M Leone S di Cianni S . A prospective, randomized comparison between ultrasound and nerve stimulation guidance for multiple injection axillary brachial plexus block. Anesthesiology. (2007) 106:992–6. doi: 10.1097/01.anes.0000265159.55179.e1, PMID: 17457131

[31] Jæger P Nielsen ZJK Henningsen MH Hilsted KL Mathiesen O Dahl JB. Adductor canal block versus femoral nerve block and quadriceps strength: a randomized, double-blind, placebo-controlled, crossover study in healthy volunteers. Anesthesiology. (2013) 118:409–15. doi: 10.1097/ALN.0b013e318279fa0b 23241723

[32] Borgeat A Ekatodramis G Dumont C. An evaluation of the infraclavicular block via a modified approach of the raj technique. Anesth Analg. (2001) 93:436–41. doi: 10.1213/00000539-200108000-00040, PMID: 11473876

[33] Song K-X Wang J-X Huang D. Therapy-induced senescent tumor cells in cancer relapse. J Nat Cancer Center. (2023) 3:273–8. doi: 10.1016/j.jncc.2023.09.001, PMID: 39036667 PMC11256611

[34] JENSTRUP MT JÆGER P LUND J FOMSGAARD JS BACHE S MATHIESEN O . Effects of adductor-canal-blockade on pain and ambulation after total knee arthroplasty: a randomized study. Acta Anaesthesiol Scand. (2012) 56:357–64. doi: 10.1111/j.1399-6576.2011.02621.x 22221014

[35] Abdel-Rehim M. New trend in sample preparation: on-line microextraction in packed syringe for liquid and gas chromatography applications. I. Determination of local anaesthetics in human plasma samples using gas chromatography-mass spectrometry. J Chromatogr B Anal Technol Biomed Life Sci. (2004) 801:317–21. doi: 10.1016/j.jchromb.2003.11.042, PMID: 14751801

[36] LUND J JENSTRUP MT JAEGER P SØRENSEN AM DAHL JB. Continuous adductor-canal-blockade for adjuvant post-operative analgesia after major knee surgery: preliminary results. Acta Anaesthesiol Scand. (2011) 55:14–9. doi: 10.1111/j.1399-6576.2010.02333.x, PMID: 21039357

[37] Grevstad U Mathiesen O Valentiner LS Jaeger P Hilsted KL Dahl JB. Effect of adductor canal block versus femoral nerve block on quadriceps strength, mobilization, and pain after total knee arthroplasty: a randomized, blinded study. Reg Anesth Pain Med. (2015) 40:3–10. doi: 10.1097/AAP.0000000000000169, PMID: 25376972

[38] Ren B Ren X Wang L Tu C Zhang W Liu Z . A bibliometric research based on hotspots and frontier trends of denosumab. Front Pharmacol. (2022) 13:929223. doi: 10.3389/fphar.2022.929223, PMID: 36199692 PMC9527327

[39] Knudsen K Beckman Suurküla M Blomberg S Sjövall J Edvardsson N. Central nervous and cardiovascular effects of i.v. infusions of ropivacaine, bupivacaine and placebo in volunteers. Br J Anaesth. (1997) 78:507–14. doi: 10.1093/bja/78.5.507, PMID: 9175963

[40] Fuster V. Impact factor: a curious and capricious metric. J Am Coll Cardiol. (2017) 70:1530–1. doi: 10.1016/j.jacc.2017.08.002 28843389

[41] Huang S-C Chiu T-M Lee C-Y Chang H-C Wu W-J Gau S-Y. Researching trends in pemphigoid diseases: a bibliometric study of the top 100 most cited publications. Front Med. (2022) 9:1088083. doi: 10.3389/fmed.2022.1088083 PMC9868262 36698818

[42] Kerr DR Kohan L. Local infiltration analgesia: a technique for the control of acute postoperative pain following knee and hip surgery: a case study of 325 patients. Acta Orthop. (2008) 79:174–83. doi: 10.1080/17453670710014950, PMID: 18484242

[43] McDonnell JG Curley G Carney J Benton A Costello J Maharaj CH . The analgesic efficacy of transversus abdominis plane block after cesarean delivery: a randomized controlled trial. Anesth Analg. (2008) 106:186–91. doi: 10.1213/01.ane.0000290294.64090.f3 18165577

[44] Liu SS Richman JM Thirlby RC Wu CL. Efficacy of continuous wound catheters delivering local anesthetic for postoperative analgesia: a quantitative and qualitative systematic review of randomized controlled trials. J Am Coll Surg. (2006) 203:914–32. doi: 10.1016/j.jamcollsurg.2006.08.007, PMID: 17116561

[45] Murouchi T Iwasaki S Yamakage M. Quadratus lumborum block: analgesic effects and chronological ropivacaine concentrations after laparoscopic surgery. Reg Anesth Pain Med. (2016) 41:146–50. doi: 10.1097/AAP.0000000000000349 26735154

[46] Blanco R Ansari T Riad W Shetty N. Quadratus Lumborum block versus transversus abdominis plane block for postoperative pain after cesarean delivery: a randomized controlled trial. Reg Anesth Pain Med. (2016) 41:757–62. doi: 10.1097/AAP.0000000000000495, PMID: 27755488

[47] Dam M Moriggl B Hansen CK Hoermann R Bendtsen TF Børglum J. The pathway of Injectate spread with the transmuscular quadratus lumborum block: a cadaver study. Anesth Analg. (2017) 125:303–12. doi: 10.1213/ANE.0000000000001922, PMID: 28277325

[48] Forero M Adhikary SD Lopez H Tsui C Chin KJ. The erector spinae plane block: a novel analgesic technique in thoracic neuropathic pain. Reg Anesth Pain Med. (2016) 41:621–7. doi: 10.1097/AAP.0000000000000451 27501016

[49] Chin KJ Malhas L Perlas A. The erector spinae plane block provides visceral abdominal analgesia in bariatric surgery: a report of 3 cases. Reg Anesth Pain Med. (2017) 42:372–6. doi: 10.1097/AAP.0000000000000581, PMID: 28272292

[50] Ivanusic J Konishi Y Barrington MJ. A cadaveric study investigating the mechanism of action of erector spinae blockade. Reg Anesth Pain Med. (2018) 43:567–71. doi: 10.1097/AAP.0000000000000789, PMID: 29746445

[51] Abdallah FW Dwyer T Chan VW Niazi AU Ogilvie-Harris DJ Oldfield S . IV and Perineural Dexmedetomidine similarly prolong the duration of analgesia after Interscalene brachial plexus block: a randomized, three-arm, triple-masked, placebo-controlled trial. Anesthesiology. (2016) 124:683–95. doi: 10.1097/ALN.0000000000000983, PMID: 26649424

[52] Vorobeichik L Brull R Abdallah FW. Evidence basis for using perineural dexmedetomidine to enhance the quality of brachial plexus nerve blocks: a systematic review and meta-analysis of randomized controlled trials. Br J Anaesth. (2017) 118:167–81. doi: 10.1093/bja/aew411, PMID: 28100520

[53] Fritsch G Danninger T Allerberger K Tsodikov A Felder TK Kapeller M . Dexmedetomidine added to ropivacaine extends the duration of interscalene brachial plexus blocks for elective shoulder surgery when compared with ropivacaine alone: a single-center, prospective, triple-blind, randomized controlled trial. Reg Anesth Pain Med. (2014) 39:37–47. doi: 10.1097/AAP.0000000000000033, PMID: 24317234

[54] Ngan Kee WD Ng FF Khaw KS Tang SPY Koo AGP. Dose-response curves for intrathecal bupivacaine, Levobupivacaine, and Ropivacaine given for labor analgesia in nulliparous women. Reg Anesth Pain Med. (2017) 42:788–92. doi: 10.1097/AAP.0000000000000657, PMID: 28991063

[55] Gong X Dan J Li F Wang L. Suppression of mitochondrial respiration with local anesthetic ropivacaine targets breast cancer cells. J Thorac Dis. (2018) 10:2804–12. doi: 10.21037/jtd.2018.05.21, PMID: 29997943 PMC6006047

[56] Polley LS Columb MO Naughton NN Wagner DS van de Ven CJM. Relative analgesic potencies of ropivacaine and bupivacaine for epidural analgesia in labor: implications for therapeutic indexes. Anesthesiology. (1999) 90:944–50. doi: 10.1097/00000542-199904000-00003, PMID: 10201661

[57] Qin Y Chen S Zhang Y Liu W Lin Y Chi X . A bibliometric analysis of endoscopic sedation research: 2001-2020. Front Med. (2021) 8:775495. doi: 10.3389/fmed.2021.775495 PMC8761812 35047526

[58] Wu H Li Y Tong L Wang Y Sun Z. Worldwide research tendency and hotspots on hip fracture: a 20-year bibliometric analysis. Arch Osteoporos. (2021) 16:73. doi: 10.1007/s11657-021-00929-2, PMID: 33866438

[59] Marhofer P Sitzwohl C Greher M Kapral S. Ultrasound guidance for infraclavicular brachial plexus anaesthesia in children. Anaesthesia. (2004) 59:642–6. doi: 10.1111/j.1365-2044.2004.03669.x, PMID: 15200537

[60] Albrecht E Kern C Kirkham KR. A systematic review and meta-analysis of perineural dexamethasone for peripheral nerve blocks. Anaesthesia. (2015) 70:71–83. doi: 10.1111/anae.12823 25123271

[61] Desmet M Braems H Reynvoet M Plasschaert S van Cauwelaert J Pottel H . I.V. And perineural dexamethasone are equivalent in increasing the analgesic duration of a single-shot interscalene block with ropivacaine for shoulder surgery: a prospective, randomized, placebo-controlled study. Br J Anaesth. (2013) 111:445–52. doi: 10.1093/bja/aet109, PMID: 23587875

[62] Choi S Rodseth R McCartney CJ. Effects of dexamethasone as a local anaesthetic adjuvant for brachial plexus block: a systematic review and meta-analysis of randomized trials. Br J Anaesth. (2014) 112:427–39. doi: 10.1093/bja/aet417, PMID: 24413428

